# Oleanolic Acid Induces the Type III Secretion System of *Ralstonia solanacearum*

**DOI:** 10.3389/fmicb.2015.01466

**Published:** 2015-12-22

**Authors:** Dousheng Wu, Wei Ding, Yong Zhang, Xuejiao Liu, Liang Yang

**Affiliations:** ^1^Laboratory of Natural Products Pesticides, College of Plant Protection, Southwest UniversityChongqing, China; ^2^Research Center of Bioenergy and Bioremediation, College of Resources and Environment, Southwest UniversityChongqing, China

**Keywords:** *R. solanacearum*, type III secretion system, plant-derived compounds, oleanolic acid, induction

## Abstract

*Ralstonia solanacearum*, the causal agent of bacterial wilt, can naturally infect a wide range of host plants. The type III secretion system (T3SS) is a major virulence determinant in this bacterium. Studies have shown that plant-derived compounds are able to inhibit or induce the T3SS in some plant pathogenic bacteria, though no specific T3SS inhibitor or inducer has yet been identified in *R. solanacearum*. In this study, a total of 50 different compounds were screened and almost half of them (22 of 50) significantly inhibited or induced the T3SS expression of *R. solanacearum*. Based on the strong induction activity on T3SS, the T3SS inducer oleanolic acid (OA) was chosen for further study. We found that OA induced the expression of T3SS through the HrpG-HrpB pathway. Some type III effector genes were induced in T3SS inducing medium supplemented with OA. In addition, OA targeted only the T3SS and did not affect other virulence determinants. Finally, we observed that induction of T3SS by OA accelerated disease progress on tobacco. Overall our results suggest that plant-derived compounds are an abundant source of *R. solanacearum* T3SS regulators, which could prove useful as tools to interrogate the regulation of this key virulence pathway.

## Introduction

*Ralstonia solanacearum* is a Gram-negative soil-borne bacterial plant pathogen that infects more than 200 plant species from over 50 families, including agriculturally and economically important crops such as tobacco, potato, eggplant, and tomato, in tropical and subtropical regions of the world (Genin, 2010). Bacterial wilt caused by this bacterium leads to serious economic losses to agricultural production every year. *R. solanacearum* enters host roots from the soil, colonizes the plant vasculature, and produces a large amount of extracellular polysaccharides (EPS), resulting in the wilting and death of host plants (Schell, 2000).

Like many other plant and animal pathogenic bacteria, *R. solanacearum* depends on the type III secretion system (T3SS) to invade its hosts. This specialized needle-like delivery machine, encoded by a cluster of about 20 hypersensitive response and pathogenicity (*hrp*) genes, can inject effector proteins directly into host cells suppressing plant innate immunity or activating effector-triggered immunity (Cunnac et al., 2004; Jones and Dangl, 2006). In *R. solanacearum*, the regulatory cascade linking the expression of T3SS to host contact has been well characterized. The initial activation of *hrp* genes is triggered upon recognition of a cell wall component by the outer membrane receptor PrhA, which transfers the plant cell interaction dependent signals through a complex signal cascade PrhA-PrhR/PrhI-PrhJ-HrpG (Brito et al., 1999, 2002; Aldon et al., 2000). HrpG, a two-component response regulator of the OmpR subfamily, is a key regulator in this cascade. Transcription of HrpG is regulated by both the above well-studied pathway and a PhcA-dependent pathway (Brito et al., 1999; Genin et al., 2005). Downstream of the central regulator HrpG is HrpB, an AraC- family transcriptional activator, which directly controls transcription of the T3SS structural genes and a large repertoire of effector genes (Occhialini et al., 2005). Another regulator that affects the transcription of the HrpB regulon is PrhG and the PrhG-HrpB pathway is regulated by an unrelated virulence operon (Plener et al., 2010; Zhang et al., 2013). Homologs of HrpB and HrpG can be found in *Xanthomonas* sp. (Wengelnik and Bonas, 1996), while other upper regulators in this cascade are not conserved in other bacterial pathogens.

Since the assembly of the T3SS and the secretion of effector proteins require a lot of energy, pathogens often do not fully express the T3SS until they come into contact with host tissues. T3SS genes in *R. solanacearum* are specifically induced in response to the bacterium-plant cell contact (Aldon et al., 2000). Recent studies also showed that the T3SS regulator *hrpB* and some *hrpB*-regulated genes are induced in bacteria growing inside infected plants (Jacobs et al., 2012; Monteiro et al., 2012), which is inconsistent with the previous view that *R. solanacearum* T3SS genes are only activated during the early stages of host recognition and bacterial colonization. On the other hand, the expression of T3SS associated genes is regulated by a variety of environmental factors (e.g., pH; growth phase; temperature; nutrition; or cell density) in different ways in different bacteria (Arlat et al., 1992; Wei et al., 1992; Van Dijk et al., 1999; Tang et al., 2006; Stauber et al., 2012). For example, the expression level of T3SS in bacteria is modulated by the carbon source. The best carbon source for *R. solanacearum hrp* genes expression is pyruvate (Arlat et al., 1992), while the optimal carbon inducer for *Pseudomonas syringae* pathovar tomato DC3000 is fructose (Stauber et al., 2012). Moreover, bacterial T3SS genes are almost inhibited in rich medium but significantly induced in minimal or inducing medium.

In addition to host signals and environmental factors, T3SS genes are also regulated by chemical or natural compounds, which can alter T3SS expression in both animal and plant pathogenic bacteria (Felise et al., 2008; Aiello et al., 2010; Duncan et al., 2012; Yang et al., 2014). This makes the T3SS an attractive target for the development of new agents for disease control. A large-scale screening has identified several classes of T3SS inhibitors, including salicylidene acylhydrazides and thiazolidinone, in animal pathogens (Bailey et al., 2007; Dahlgren et al., 2007; Negrea et al., 2007; Felise et al., 2008; Tree et al., 2009; Veenendaal et al., 2009). Thiazolidinone has further been shown to block the T3SS of the plant pathogen *P. syringae* (Felise et al., 2008). Recently, some plant phenolic compounds and their derivatives (e.g., *p*-coumaric acid; benzoic acid; *trans*-4-hydroxycinnamohydroxamic) were found to be able to inhibit the T3SS in plant pathogens *Erwinia amylovora* or *Dickeya dadantii* (Li et al., 2009, 2015; Khokhani et al., 2013). Interestingly, some T3SS inducers [e.g., *o*-coumaric acid; *t*-cinnamic acid; *trans*-2-(4-hydroxyphenyl)-ethenylsulfonate] were also found for *E. amylovora* or *D. dadantii* (Yang et al., 2008; Khokhani et al., 2013). These phenolic compounds inhibit or induce the T3SS through different signal pathways, even in the same phytopathogenic bacteria. For example, the inhibition of *D. dadantii* T3SS expression by *p*-coumaric acid is moderated through the HrpX/Y two-component system (Li et al., 2009), while *o*-coumaric acid and *t*-cinnamic acid induce the *D. dadantii* T3SS expression through the *rsmB*-RsmA pathway (Yang et al., 2008). Furthermore, the inhibition of T3SS in *E. amylovora* by exogenous compounds could reduce disease development and T3SS dependent HR (Khokhani et al., 2013; Yang et al., 2014), suggesting it is possible to control plant diseases through the inhibition of T3SS. Although some efforts have been put into the identification of T3SS inhibitors or inducers for plant pathogens, regulation of the T3SS by plant-derived compounds remains largely unknown in most plant pathogenic bacteria.

Given that the T3SS is well conserved in animal and plant pathogenic bacteria, and is able to be either repressed or induced by exogenous compounds in some specific pathogens, we hypothesized that some compounds may alter the expression of T3SS in *R. solanacearum*. In this study, different kinds of compounds were screened for their effect on *R. solanacearum* T3SS expression. The results showed that T3SS expression of this bacterium was either induced or inhibited by some of the screened compounds. Oleanolic acid (OA), one of the best inducers, was further evaluated for its effect on T3SS regulatory components. Furthermore, the effect of OA on disease development in tobacco was investigated.

## Materials and Methods

### Bacterial Strains and Growth Conditions

The *R. solanacearum* wild-type strain CCT818 (phylotype I, race 1, biovar 3) and the *popA-lacZYA* reporter strain CCT877 were used in this study. The wild-type strain was originally isolated from an infected tobacco plant in Chongqing, China in 2013. The *popA-lacZYA* reporter strain was constructed using the recombinant plasmid ppop3 as previously described (Zhang et al., 2011). *R. solanacearum* was grown in rich B medium or hydroponic plant culture medium supplemented with 2% sucrose [plant-sucrose (PS) medium] at 28°C (Yoshimochi et al., 2009).

### Sources and Description of Screened Compounds

Compounds 1–14, 16–38, and 47–50 were purchased from Shanghai Yuanye Bio-Technology Co., Ltd (Shanghai, China). Compound 15 was extracted and purified from the leaves of *Artemisia annua* L by our lab members. Compounds 39 to 46 were purchased from Sangon Biotech (Shanghai, China). Among them, compounds 1–38 are natural or plant-derived compounds, which have been reported to have good biological activity in medicine or as pesticides in the field. Compounds 39–46 are major root exudates of tobacco. Compound 47 was previously reported to inhibit T3SS in *E. amylovora* (Khokhani et al., 2013) and compound 48 was reported to induce the T3SS genes of the plant pathogen *D. dadantii* (Yang et al., 2008). Compounds 49 and 50 are widely used plant disease resistance inducers. When screening, compounds were first resolved in dimethyl sulfoxide (DMSO) and a final concentration of 100 μM was added to the PS medium.

### β-Galactosidase Activity Assay Based Compounds Screening

Expression of *popA* was analyzed by determining the β-galactosidase activity of the *lac-ZYA* reporter gene. The β-galactosidase activity assay was performed as previously described with appropriate modifications (Zhang et al., 2015). The *popA-lacZYA* reporter strain was grown in rich B broth overnight and transferred to *hrp*-inducing PS medium supplemented with DMSO or 100 μM concentrations of the compounds. PS medium was used to mimic the plant environment and induce the *hrp* genes expression. When OD_600_ of the culture suspension reached 0.1 to 0.2, the β-galactosidase activity of each treatment were measured. Enzyme activities were calculated as previously described (Zhang et al., 2015). Three replicates were used in each of the independent experiment and each experiment was repeated two times.

### Measurement of Growth Curve

*R. solanacearum* cells were first grown overnight in rich B broth at 28°C. The bacterial suspension (OD600≈1.0) was then transferred into fresh B medium supplemented with DMSO or 100 μM of OA with a proportion of 1:100. The growth curve was monitored for 36 h with a 4-h interval. The cultures were grown in 250-ml culture flasks, and 3 ml of the cultures were taken at each time point to determine the OD_600_ value by using the normal spectrophotometer. Three independent experiments were performed and three culture flasks were used for each treatment in each independent experiment.

### Biofilm Assay

To measure biofilm formation in *R. solanacearum* after compounds treatment, the polyvinylchloride (PVC) microtiter plate assay was used as previously described with some modifications (Yao and Allen, 2007). Briefly, overnight cultured bacterial suspension was centrifuged and resuspended in sterile water; the OD_600_ of the resuspension was exactly adjusted to 0.1. Then 5 μl of the OD_600_ adjusted suspension was added into 95 μl of B medium supplemented with DMSO or different concentrations of OA and incubated at 30°C for 24 h without shaking. Crystal violet staining and biofilm quantification were performed as previously described (Yao and Allen, 2007) except that the absorbance was determined at 490 nm using a micro-plate reader (Bio-Rad).

### RNA Extraction and Quantitative Real-Time PCR

Overnight cultured bacterial suspension was inoculated to fresh PS medium supplemented with DMSO or 100 μM concentrations of compounds and shake culture at 28°C for 6 h. Bacterial cells were collected by centrifuge. Total RNA was extracted from the collected cells using TRNzol reagent according to the manufacturer’s instructions (Tiangen Biotech Co. Ltd, Beijing, China) and then treated with RNase-free DNase I (Tiangen Biotech Co. Ltd, Beijing, China) to remove any genomic DNA contaminations. RNA degradation and contamination were checked on 1% agarose gels and RNA concentration and purity were monitored using the Nanovue UV-Vs spectrophotometer (GE Healthcare Bio-Science, Uppsala, Sweden). cDNA was synthesized from 1 μg of total RNA using the iScript cDNA synthesis kit (Bio-Rad, Hercules, CA, USA).

The primers of tested genes were designed using Primer Blast in NCBI and synthesized by Life Technologies. Sequences of the primers are listed in **Supplementary Table S1**. The *serC* gene was used as the reference gene to normalize gene expression (Monteiro et al., 2012). All quantitative real-time PCR (qRT-PCR) analyses were performed on the CFX96 Manager (Bio-Rad) in a 20 μl reaction system which consisted of 10 μl Sso Fast ^TM^ EvaGreen Supermix (Bio-Rad), 1 μl of diluted cDNA, 0.2 mM of each primer and 7 μl of milliQ H_2_O. The amplification protocol was as follows: 3 min at 95°C, followed by 40 cycles of 95°C for 10 s, and 60°C for 20 s. After that, a melting curve from 60 to 95°C was applied to test the specificity and consistency of the PCR products. Normalized gene expression was calculated by Bio-Rad CFX Manager 3.0 software using the ΔΔCq method.

### Virulence Assay

Normal soil drenching was used to evaluate the virulence of *R. solanacearum* after OA treatment. *R. solanacearum* cells were shake culture in PS medium supplemented with DMSO or 100 μM of OA at 28°C for 6 h and bacterial suspension of each treatment was adjusted to the same concentration. Each individual unwounded 6-week old tobacco plant (Yunyan87) was soaked with either DMSO treated or OA treated bacterial suspension to create a final inoculation density of 1 × 10^8^ CFU/g soil medium. Inoculated plants were put into the climate room at 28°C with 14 h/10 h light/dark cycle. Disease was recorded daily using a disease index scale of 0–4 (0: no symptoms appeared; 1: 1–25% of leaves wilted; 2: 26–50% of leaves wilted; 3: 51–75% of leaves wilted; 4: 76–100% of leaves wilted). Each treatment contained 16 plants in an independent experiment and the inoculation assay was repeated three times.

### Statistical Analysis

Data were statistically analyzed using a one-way analysis of variance (ANOVA) and Student’s *t*-test under the significance level of 0.05 (*P*-value = 0.05) in SPSS 17.0. Disease index significance analysis was performed as previously described using repeated-measures ANOVA (Jacobs et al., 2013).

## Results

### A Screen Identifies Oleanolic Acid as a Strong Inducer of T3SS

**FIGURE 1:**
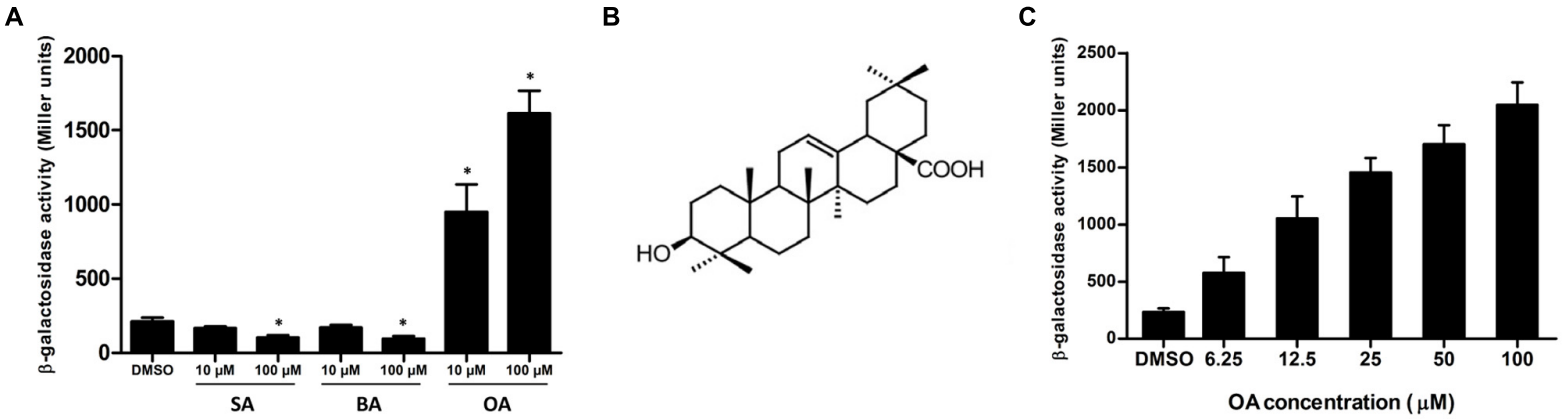
**Oleanolic acid (OA) induces T3SS expression of *Ralstonia solanacearum* in a concentration-dependent manner.** **(A)** β-galactosidase activity of the *popA-lacZYA* reporter fusion gene in plant-sucrose (PS) medium supplemented with dimethyl sulfoxide (DMSO) or PS medium supplemented with low (10 μM) or high (100 μM) concentrations of stearic acid (SA), benzoic acid (BA) and oleanolic acid (OA). Values are representative of two independent experiments. Asterisks indicate that β-galactosidase activity after compound treatment is significantly lower or higher than that of the DMSO control (*P* < 0.05, Student’s *t*-test). **(B)** Chemical structure of OA (Pollier and Goossens, 2012). **(C)** β-galactosidase activity of the *popA-lacZYA* reporter fusion gene in PS medium supplemented with DMSO or PS medium supplemented with different concentrations of OA. Columns represent the average β-galactosidase activity of three independent experiments; error bars indicate the standard deviation.

In order to screen for compounds that could induce or repress the expression of T3SS in *R. solanacearum*, a T3SS reporter strain was constructed. The *lacZYA* reporter gene which encodes a variant of β-galactosidase was inserted into the type III effector gene *popA*. In the *popA-lacZYA* fusion strain, the *lacZYA* gene shares the promoter with *popA* whose activity represents the expression level of the downstream T3SS. This reporter strain was cultured in a PS medium, which had a stronger induction on *popA* expression than *hrp*-inducing minimal medium (Yoshimochi et al., 2009), supplemented with DMSO (solvent of the screened compounds) or 100 μM of each compound. The expression level of *popA* was then determined by measuring the β-galactosidase activity (**Table 1**). Among the 50 plant-derived compounds screened, six compounds had a strong inhibitory effect on the measured *popA* promoter activity. The expression of *popA* was reduced by more than 50% of the reference level after stearic acid (compound 41, SA) and benzoic acid (compound 47, BA) treatment at the concentration of 100 μM. Since SA and BA inhibited the expression of *popA* at a high concentration, we further examined the inhibitory effect of these two compounds at a concentration of 10 μM. The result showed that 10 μM of SA and BA did not result in significant inhibition on *popA* expression (**Figure 1A**). In addition to these inhibitors, however, 16 other compounds showed a significant induction effect on *popA* expression, among which compound 28, OA (**Figure 1B**), was the best inducer with an 8.1-fold increase in β-galactosidase activity at the concentration of 100 μM compared to DMSO control. A strong induction effect was also observed when OA was used at a concentration of 10 μM (**Figure 1A**). Although we were initially interested in screening for strong T3SS inhibitor(s), we found that in our experiments OA was a more attractive compound for investigating the regulatory mechanism of *R. solanacearum* T3SS by exogenous compounds because of the strong induction effect.

**Table 1:** Expression of *Ralstonia solanacearum popA* measured by the β-galactosidase activity of the *lacZYA* reporter fusion gene in PS medium or PS medium supplemented with plant derived compounds.

Number	Compound^a^	β-galactosidase activity (mean ± SD)^b^	Number	Compound^a^	β-galactosidase activity (mean ± SD)^b^
	DMSO	368 ± 5.2		DMSO	194 ± 10.0
(1)	Chlorogenic acid	661 ± 17.2ˆ*	(26)	Synephrine	224.5 ± 7.5
(2)	Protocatechuic acid	430 ± 2.5ˆ*	(27)	Curcumol	185 ± 17.0
(3)	Muscone	388 ± 21.9	(28)	Oleanolic acid	1580 ± 34.5ˆ*
(4)	Resveratrol	467 ± 4.7ˆ*	(29)	Arctigenin	221 ± 9.5
(5)	Eugenol	350 ± 1.0	(30)	Dicoumarolum	236 ± 19.5
(6)	Gastrodin	363 ± 3.3	(31)	Luteolin	282 ± 62.0
(7)	Cepharanthine	431 ± 13.4ˆ*	(32)	Esculin hydrate	275 ± 6.8ˆ*
(8)	Deoxyschizandrin	394 ± 14.5	(33)	Apigenin	462 ± 10.0ˆ*
(9)	Capsaicin	434 ± 9.5ˆ*	(34)	Scopolamine butylbromide	207 ± 7.5
(10)	Icariin	247 ± 6.1ˆ*	(35)	Diosgenin	180 ± 7.0
(11)	Tetrandrine	410 ± 28.0	(36)	Daphnetin	204 ± 8.5
(12)	Alantolactone	362 ± 6.7	(37)	Stigmasterol	201 ± 10.5
(13)	Palmitic acid	284 ± 14.9ˆ*	(38)	Glycyrrhetic acid	156 ± 2.0ˆ*
(14)	Citric acid	425 ± 52.8			
				DMSO	186 ± 11.0
	DMSO	310 ± 4.0	(39)	Succinic acid	259 ± 5.0ˆ*
(15)	Scopoletin	452 ± 31.5ˆ*	(40)	Fumaric acid	277 ± 13.5ˆ*
(16)	Tubeimoside	375 ± 8.5ˆ*	(41)	Stearic acid	86 ± 9.5ˆ*
(17)	Xanthotoxin	324 ± 24.5	(42)	L-Malic acid	396 ± 37.5ˆ*
(18)	Bergeninum	352 ± 4.0ˆ*	(43)	L-Tryptophan	190 ± 14.5
(19)	Lappaconitine	314 ± 13.0	(44)	L-Histidine	194 ± 10.5
(20)	Taxifolin	786 ± 28.0ˆ*	(45)	L-valine	211 ± 16.0
(21)	Podophyllotoxin	389 ± 3.5ˆ*	(46)	L-Arginine	212 ± 5.5
(22)	Cryptotanshinone	344 ± 56.5	(47)	Benzoic acid	73 ± 8.5ˆ*
(23)	Vindoline	319 ± 32.0	(48)	Trans-Cinnamic acid	164 ± 8.0
(24)	Emodin	214 ± 12.5ˆ*	(49)	Methyl Jasmonate	193 ± 9.6
(25)	Patchouli alcohol	365 ± 24.5	(50)	Salicylic acid	212 ± 11.0


*^a^PS medium (plant-sucrose medium) was supplemented with DMSO or 100 μM of the indicated compounds. The compounds were assayed two times.*

*^b^*Ralstonia solanacearum* strain carrying *popA-lacZYA* reporter was used in this experiment. The promoter activities of bacteria culture fluid with OD_600_ of 0.1–0.2 were determined. Values are representative of two independent experiments and three replicates were used for each experiment. Asterisks indicate statistically significantly differences in *popA* expression between bacterial cells grown in PS medium supplemented with DMSO and PS medium supplemented with different compounds (*P* < 0.05, Student’s t-test).*

### OA Induces the Expression of T3SS in a Concentration-Dependent Manner

To further determine the effect of the OA concentration on *popA* expression, we examined the induction effect of this compound at different concentrations, ranging from 6.25 to 100 μM. Consistent with the initial screening, 100 μM of OA showed an 8.8-fold induction compared to the DMSO control (**Figure 1C**). The fold induction of *popA* by OA at concentrations of 50, 25, and 12.5 μM was 7.3, 6.3, and 4.5, respectively. The expression of *popA* was induced by a 2.5-fold of level when 6.25 μM of OA was supplemented in the PS medium. The induction activity was decreased with the reduced OA concentration, suggesting that OA induced *popA* expression in a concentration-dependent manner.

### OA Induces T3SS Expression Through the HrpG-HrpB Pathway

**FIGURE 2:**
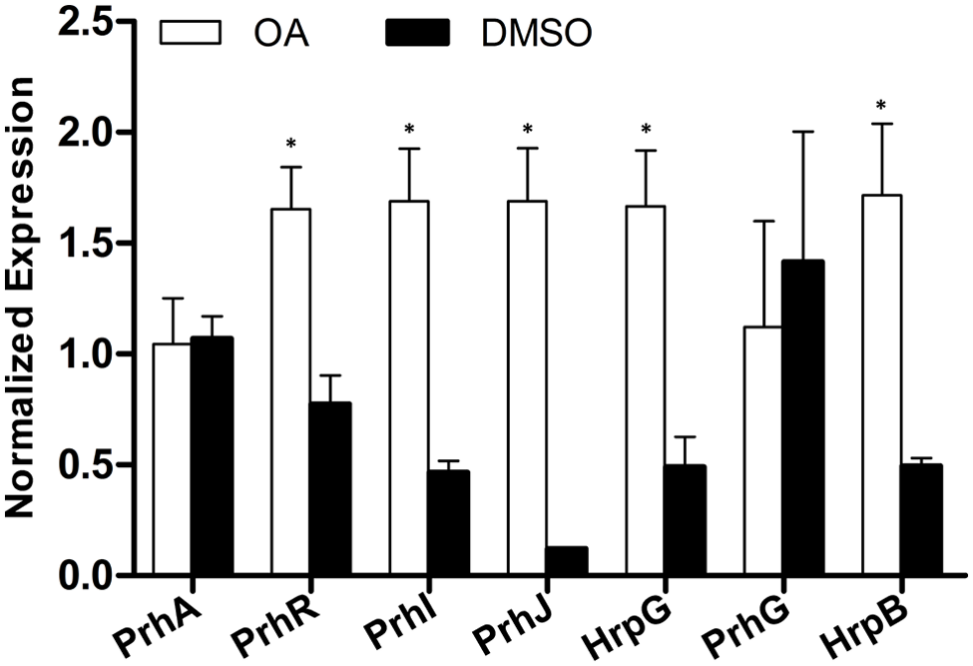
**Some but not all type III effector genes are induced by OA.** The relative expression level of nine representative type III effector genes in PS medium supplemented with DMSO or 100 μM of OA were determined by qRT-PCR. *SerC* was used as the reference gene to normalize the type III effector gene expression using the ΔΔCq method. Results are the average value of two biological replicates; error bars indicate the standard deviation. Asterisks indicate that these effector genes are significantly induced by OA compared to the DMSO control (*P* < 0.05, Student’s *t*-test).

In the previous experiments, we used a *popA-lacZYA* reporter strain to measure *popA* expression and we found that OA induced the expression level of *popA*. In order to confirm this finding, we measured the mRNA level of *popA* after OA treatment by qRT-PCR. Compared to the DMSO control, a significantly higher level of *popA* mRNA was observed when the PS medium was supplemented with 100 μM of OA (**Figure 2**). This result was consistent with the previous reporter strain based assay that showed that *popA* expression in *R. solanacearum* was induced by OA.

**FIGURE 3:**
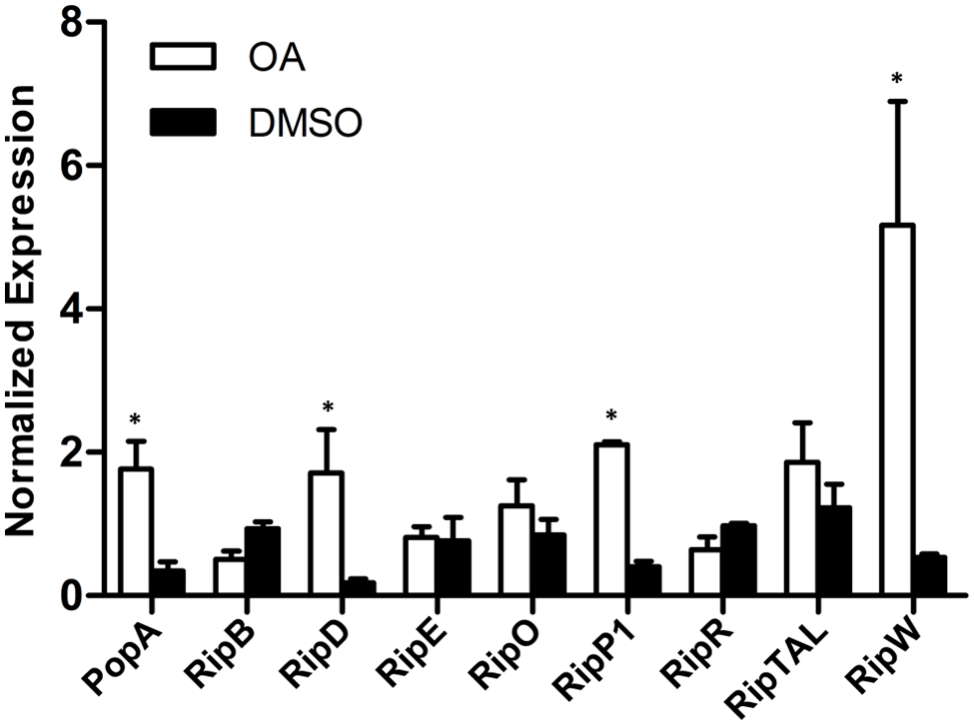
**Induction of *R. solanacearum* T3SS by OA is through the HrpG-HrpB pathway.** qRT-PCR was used to measure expression of T3SS pathway genes in PS medium supplemented with DMSO or 100 μM of OA. *SerC* was used as the reference gene to normalize the gene expression using the ΔΔCq method. These results reflect two biological replicates and error bars indicate the standard deviation. Asterisks indicate statistically significantly differences in gene expression between bacterial cells grown in PS medium supplemented with DMSO and PS medium supplemented with 100 μM of OA (*P* < 0.05, Student’s *t*-test).

*popA* is a type III effector gene and its expression is directly controlled by HrpB whose expression is further regulated by HrpG, PrhG and other upstream regulators. Since the induction effect of OA on *popA* expression has been validated, we want to know the effect of OA on the regulatory components of the T3SS which have been well described in *R. solanacearum* (Peeters et al., 2013b). The mRNA levels of genes in the T3SS signal pathway after DMSO or OA treatment were then measured. Our result showed that most of the T3SS upstream regulators were induced upon addition of 100 μM of OA when compared to the DMSO control (**Figure 3**). The expression level of *PrhG* was not significantly affected after OA treatment. This suggests that the T3SS inducer OA induces the T3SS expression through the HrpG-HrpB pathway.

### OA Induces Only a Subset of Type III Effector Genes

In *R. solanacearum*, HrpB is a downstream regulator in the T3SS signal cascade and it directly controls the transcription of type III effector genes. Based on the initial experiments, we found that OA altered the expression of both the T3SS downstream regulation gene *hrpB* and the type III effector gene *popA*. To determine whether the induction of *hrpB* by OA results in transcription activation of other effector genes, qRT-PCR was performed to examine the mRNA levels of other effector genes in the presence and absence of OA. Because *R. solanacearum* has a large repertoire of effectors and it is quite difficult to test the expression of all other effector genes, we chose eight representative effector genes for this experiment. The result showed that five effector genes were similarly expressed in DMSO or OA treated cells (**Figure 2**). However, significantly higher levels of mRNA of another three effector genes, *RipD*, *RipP1*, and *RipW*, were observed when the PS medium was supplemented with 100 μM OA. This indicates that only a subset of type III effector genes were induced by the T3SS inducer OA.

### Other Virulence Factors in *R. solanacearum* are not Affected by OA

We hypothesize that T3SS inducers or inhibitors target only the bacterial T3SS, but not other virulence factors that play important roles in different infection stages. To test this, the growth rate of *R. solanacearum* after OA treatment was first determined. Since PS medium used for T3SS induction is a nutrition-poor medium and not able to support the growth of *R. solanacearum* over an OD_600_ of 0.2, B medium was selected to test the effect of OA on bacterial growth. In a 36 h growth curve, 100 μM of OA did not cause a significant induction or inhibition on the growth of the bacterium (**Supplementary Figure S1**). Having shown that OA does not affect the *R. solanacearum* growth, we further tested whether this compound affects biofilm formation. A standard PVC microtiter plate assay was used to quantify *R. solanacearum* biofilm formation. The result showed that there was no significant difference in bacterial biofilm formation after treatment with DMSO or different concentrations of OA treatment (**Supplementary Figure S2**).

In *R. solanacearum*, the T3SS is linked with the quorum sensing system and the type II secretion system by the global virulence regulator PhcA (Mole et al., 2007). Given that OA significantly induced the expression of T3SS genes, we were interested in the effect of OA on the expression of other virulence regulation genes. To test this, we measured the mRNA level of the key regulator PhcA; quorum sensing regulators PhcB, PhcR, and PhcS; and EPS secretion related genes *XpsR* and *EpsE*. Although OA reduced the expression of *PhcA* and several other genes, no significant inhibition was observed when evaluated with significance analysis at the level of *p* = 0.05 (**Supplementary Figure S3**). It seems that OA does not affect the expression of other virulence regulation genes.

### OA Accelerates Disease Progress of Bacterial Wilt on Tobacco

**FIGURE 4:**
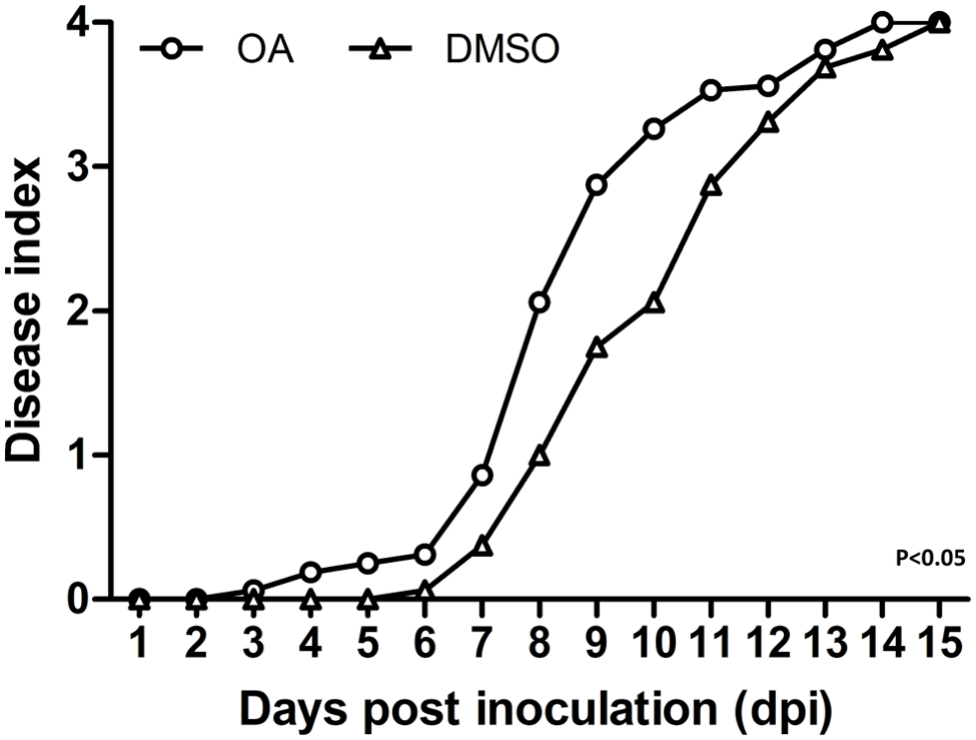
**Induction of T3SS by OA accelerates disease progress on tobacco.** 6-week-old tobacco plants were inoculated with *R. solanacearum* treated with DMSO or 100 μM of OA by pouring a bacterial suspension onto a pot containing an unwounded plant. Symptoms were rated daily using a disease index scale of 0–4 (0, no symptoms appeared; 1, 1–25% of leaves wilted; 2, 26–50% of leaves wilted; 3, 51–75% of leaves wilted; 4, 76–100% of leaves wilted). Each point represents the average disease index of 16 plants. The OA treatment was significantly different from the DMSO treatment (*P* < 0.05; repeated-measures ANOVA). Similar results were observed in other two independent experiments.

Based on the strong induction effect of OA on *R. solanacearum* T3SS, its ability to accelerate bacterial wilt disease progress was evaluated. In order to mimic the natural infection process, a soil soak virulence assay was used to measure the wilt disease progress of DMSO or OA treated bacteria on tobacco Yunyan87, the cultivar from which the original wild-type strain was isolated. Compared to the DMSO control, OA treated *R. solanacearum* accelerated the wilting of inoculated plants [*P* < 0.05; repeated-measures analysis of variance (ANOVA)] (**Figure 4**). This experiment suggests that OA-mediated T3SS induction makes the bacteria more virulent on host plants.

## Discussion

In this study, a total of 50 plant-derived compounds were tested for their activity on inhibiting or inducing the *R. solanacearum* T3SS expression. We found that 22 compounds significantly inhibited or induced the expression of the T3SS in this bacterium (**Table 1**), suggesting that plant-derived compounds are an abundant source of exogenous regulators of *R. solanacearum* T3SS. We were initially looking for T3SS inhibitors for this devastating plant pathogen and we did find some compounds (e.g., BA and SA) that are able to reduce the T3SS expression of this bacterium. However, the inhibitory effect of these compounds was not so pronounced. But interestingly, the compound OA strongly induced T3SS expression of *R. solanacearum*. Based on this observation, OA was chosen as an interesting candidate with which to investigate the mechanism of *R. solanacearum* T3SS regulation by exogenous compounds. Further experiments demonstrated that OA induced the T3SS through the HrpG-HrpB pathway and activated expression of some, but not all, type III effector genes. This T3SS inducer targeted only the T3SS without affecting other virulence determinants. In addition, induction of *R. solanacearum* T3SS by OA accelerated disease progress on tobacco.

Oleanolic acid is a widely distributed pentacyclic triterpenoid compound throughout the plant kingdom with several promising pharmacological activities, such as being an anti-inflammatory and antioxidant (Pollier and Goossens, 2012). It is interesting that OA was found to be able to induce T3SS expression of *R. solanacearum* in this study, suggesting that this special compound has a variety of biological functions in nature. Although T3SS induction by OA is beneficial for the pathogen, which does not comply with the promising pharmacological activity of OA, we here just regard it as a representative compound to investigate the mechanism of *R. solanacearum* T3SS regulation by exogenous compounds. It is in fact, not a surprise that the *R. solanacearum* T3SS was induced by OA. It has been shown that when pyruvate was supplemented as the carbon source in the medium, the level of *R. solanacearum* T3SS expression was induced (Arlat et al., 1992). More, previous studies have identified several plant phenolic compounds and derivatives as the T3SS inducers of *D. dadantii*, *P. aeruginosa* and *E. amylovora* (Yang et al., 2008; Yamazaki et al., 2012; Khokhani et al., 2013). These studies suggest that it is possible to induce the expression of the T3SS in diverse bacterial pathogens by plant-derived compounds, which is consistent with our finding that the T3SS of *R. solanacearum* could be induced by OA. Some of the identified T3SS inducers are shown to have higher induction activity when used at high concentrations. In agreement with these observations, our data also showed that OA induced *R. solanacearum* T3SS expression in a concentration-dependent manner (**Figure 1C**). Since OA is a kind of plant-derived compound, we speculate that OA mimics the host environment and thus activates the T3SS expression. If this assumption is true, it may open new directions in chemical level for host–microbe interaction studies. That means, during the compatible interaction between susceptible hosts and pathogens, the host may secrete some compounds to induce the T3SS expression and favor the pathogen invasion. If it is true, identifying such compounds will deepen our current understanding of the host–microbe interaction.

**FIGURE 5:**
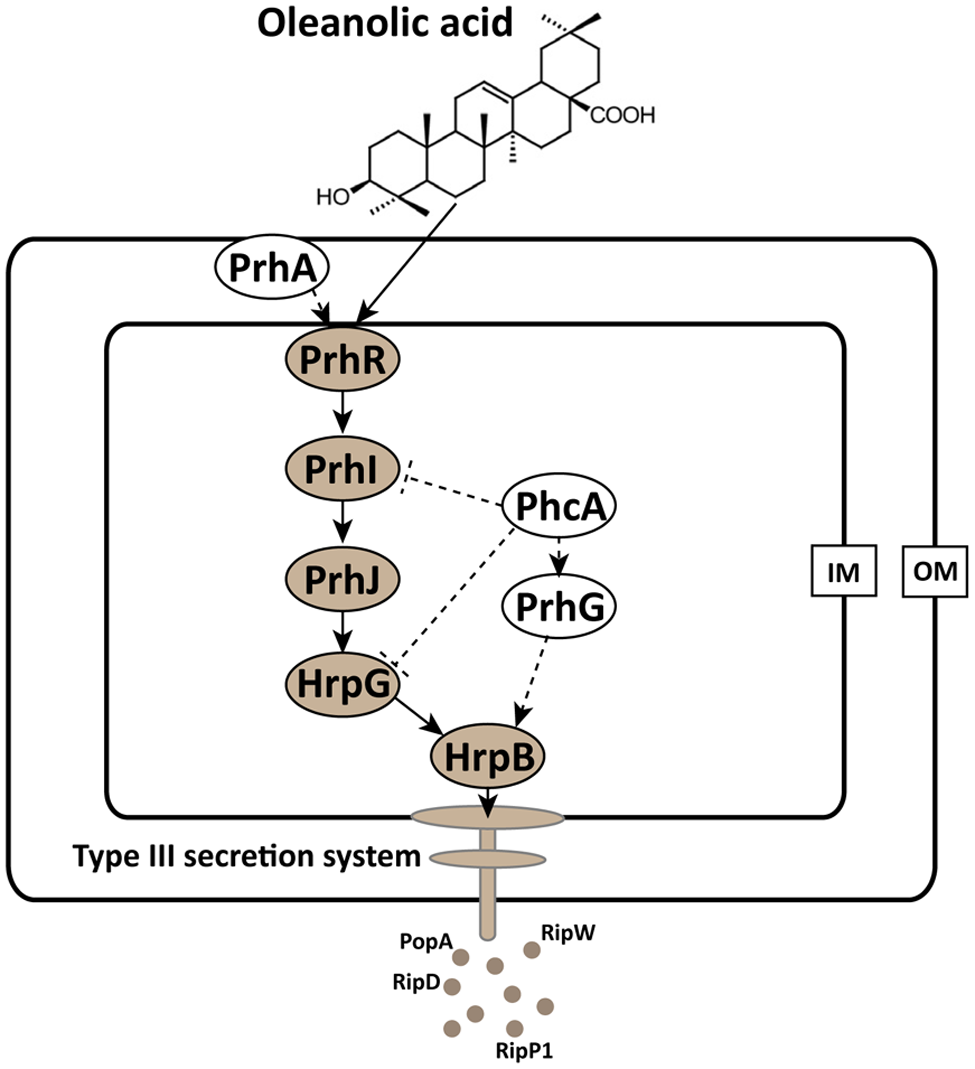
**Mode of OA on T3SS induction in *R. solanacearum*.** The *hrp* gene expression in *R. solanacearum* is directly controlled by HrpB. HrpB is further controlled by two independent cascades PchA-PrhG and PrhA-PrhR/I-PrhJ-HrpG. The global regulator PhcA can also modulate T3SS expression through PrhI and HrpG (Peeters et al., 2013b). In this study, we observed that the T3SS inducer OA induced the type III effector gene expression (e.g., *popA*, *RipD*, *RipW*, and *RipP1*) through the HrpG-HrpB pathway. Ovals with gray background indicate regulators that induced by OA. Solid line arrows indicate the direct induction of these regulators by OA. IM, inner membrane; OM, outer membrane.

The T3SS signal cascade has been well characterized in *R. solanacearum*. PrhA, an outer membrane receptor at the top of the *hrp* regulatory pathway, is responsible for perceiving plant signals and activating the downstream regulators (Marenda et al., 1998; Aldon et al., 2000). The PrhA-dependent activation of T3SS is specific to bacteria-host contact but not required in medium (Marenda et al., 1998). In this study, we found that PS medium supplemented with OA induced downstream T3SS expression, but it did not induce the expression of the top regulator PrhA (**Figure 3**). There is a possibility that OA directly activates the PrhA protein at post-translational level but not transcriptional level. PrhI, PrhR, and PrhJ, the downstream regulators of PrhA, have also been shown to activate *hrp* gene expression in response to contact with plant cells but not in the medium (Brito et al., 1999, 2002). It is worth noting that *PrhI*, *PrhR*, and *PrhJ* were induced by OA in our experiment. This suggests that although these regulators are not required for activation of *hrp* genes in medium, they can still be induced in medium. Another regulator in this cascade is HrpG, which is required for T3SS gene expression in minimal medium (Brito et al., 1999). Consistent with the previous study, our data demonstrated that *hrpG* expression was significantly induced by OA. In *R. solanacearum*, *hrp* gene expression in minimal medium is also controlled by PrhG which belongs to an independent pathway (Plener et al., 2010). In contrast with some other regulators, this regulator is required for T3SS gene activation in medium but not in the presence of plant cells. Interestingly, the medium-response regulator PrhG is not affected by OA. But PrhG showed a higher basal expression compared to other regulators. Together, our results suggest that the T3SS inducer OA affects the expression of T3SS through HrpG-HrpB pathway (**Figure 5**).

*R. solanacearum* has a large repertoire of type III effectors and homology of approximately one-third of effectors can be found in other bacterial plant pathogens (Peeters et al., 2013a; Deslandes and Genin, 2014). Although limited is known about the regulation of the effector genes expression in medium, an *in planta* transcriptome analysis has revealed that almost half of the effector genes are up-regulated in wilting tomato plants compared to expression in rich medium (Jacobs et al., 2012). This indicates firstly that type III effector production is still required at the later stages of bacterial wilt. Second, only a subset of effector genes from a given strain may contribute significantly to disease on a given host. Most importantly, the activation of HrpB does not mean that all downstream effector genes are also activated in a given environment, though HrpB is the master regulator of all the effector genes. In our study, we found that OA significantly induced HrpB, but only three of eight tested effector genes were induced by OA (**Figure 2**). The *R. solanacearum* strain that we used in this study is originally isolated from tobacco. We do not know exactly how many effector genes our strain has, but based on the previous study (Jacobs et al., 2012), we guess only a subset of them are up-regulated in infected tobacco plants. The eight effectors we chose here are only representative conserved effector genes in *R. solanacearum*. We speculate that OA induced the expression of more effector genes if we test more and these induced effectors contribute to bacterial virulence. The later inoculation assay showed that *R. solanacearum* treated with OA is more virulent than *R. solanacearum* treated with DMSO (**Figure 4**), which may support the speculation that OA enhanced bacterial virulence by inducing the expression of some effector genes.

It is known that the T3SS is a vital pathogenicity factor in bacterial pathogens. Disruption of genes encoding structural components of the T3SS or upstream regulators always results in non-pathogenicity (Boucher et al., 1987). Based on this, researchers have tried to use *hrp-* mutants that are defective in T3SS activation or translocation as potential bio-control agents to control bacterial wilt (Frey et al., 1994), but this approach failed in field conditions. However, we can see from these studies that instead of producing *hrp-* mutants, inhibiting T3SS expression by using compounds would be an alternative option. Interestingly, our study identified a T3SS inducer. We also found that induction of the T3SS by this inducer accelerated the disease progress on tobacco, suggesting that if potential T3SS inhibitors exist, the T3SS inhibitor would be able to delay or even prevent disease occurrence.

In summary, this study screened a total of 50 plant-derived compounds and identified that some of them could inhibit or induce the *R. solanacearum* T3SS expression. The mechanism of the representative T3SS inducer OA on regulation of *R. solanacearum* T3SS was further elucidated. These results suggest that plant-derived compounds are an abundant source of *R. solanacearum* T3SS regulators. Further screening may identify some strong T3SS inhibitors to disable the normal function of *R. solanacearum* T3SS providing an alternative strategy for the control of bacterial wilt.

## Author Contributions

Conceived and designed the experiments: WD performed the experiments: DW, YZ, XL, LY and WD. Analyzed the data: DW and WD wrote the paper: DW and WD.

## Conflict of Interest Statement

The authors declare that the research was conducted in the absence of any commercial or financial relationships that could be construed as a potential conflict of interest.
