# Supplementary material for: Oleanolic Acid Induces the Type III Secretion System of Ralstonia solanacearum
Source: Front Microbiol. 2015 Dec 22;6:1466. doi: 10.3389/fmicb.2015.01466 (PMC4686671; doi:10.3389/fmicb.2015.01466)
Supplement: Supplementary file 1 [file Table_1.PDF]

Table S1 Primers used in this study

Name	Sequence (5’ to 3’)	Product Size	Source
qSerc-F	CCCACCTACGCCATCTATGT		This study
qSerc-R	TTGAGGAAGAACGGCACATT	191 bp	This study
qPrhA-F	GTTGGCATCGATGTGGTACA		This study
qPrhA-R	GCGTGGTCTACAAGCTGGAC	197 bp	This study
qPrhR-F	CAGGGCATGGTGGAGGTC		This study
qPrhR-R	TATCCACCAGCATCTGCAAG	163 bp	This study
qPrhI-F	GATCTGGAGCTGGCCTACC		This study
qPrhI-R	CGCCTGGGTCATGTACTTCT	240 bp	This study
qPrhJ-F	CTGGTCGATCTGGAGCAGTT		This study
qPrhJ-R	CGATCTCCACATTGGATTTG	226 bp	This study
qHrpG-F	GTCTTCACGGTCTGCGAACT		This study
qHrpG-R	ATTGACCTCCAATCCATCCA	247 bp	This study
qPrhG-F	TCCAGATGCTGATGATCGAC		This study
qPrhG-R	AGTTCGTCCGCAGAGAAATG	193 bp	This study
qHrpB-F	AATACGCAAATGCGGTTTTC		This study
qHrpB-R	CTTCTTCCGCTTCTTCATCG	156 bp	This study
qPopA-F	AGGATCTGCTCGATCTCCTG		This study
qPopA-R	AAGACCTGGTGAAGCTGCTG	151 bp	This study
qRipB-F	GTCATCGGGTCGTACAGGTT		This study
qRipB-R	CCTGAAAGACGTCCAGAAGC	210 bp	This study
qRipD-F	GGATGTACTCCTTGGCCGTA		This study
qRipD-R	AGATGGAAACCCACAAGACG	150 bp	This study
qRipE-F	TCGACATTTCTGCTTTGACG		This study
qRipE-R	GAGAACGAAGTTCGGCTACG	215 bp	This study
qRipO-F	TCCAGGTCTTCCTGATACGG		This study
qRipO-R	CCAGCTCCTGCTGATCTACC	159 bp	This study
qRipP1-F	ACTGTGTTGCCTTTGCACTG		This study
qRipP1-R	GTTGTGGTACAGCCCTCGAT	161 bp	This study
qRipR-F	CGCGTTGATGTAGGAAGGAT		This study
qRipR-R	CGAATTCAACATCCCGAACT	190 bp	This study
qRipTAL-F	AGTTCACGCCAGATCGTCTT		This study
qRipTAL-R	ATTTGCAGAAGCCAGTCGAG	208 bp	This study
qRipW-F	AGGCCTTGTAGCTCACCTTG		This study
qRipW-R	CCAGGATTCGAATTTCCAGA	164 bp	This study
qPhcA-F	TTGTAGGTCTCGCACACCAG		This study
qPhcA-R	GCTCGCTCGATCAGTACCTC	235 bp	This study
qPhcB-F	CCAGATCGTCGTCAATGAAA		This study
qPhcB-R	TTCGAGCCAGTCGAGGTAGT	178 bp	This study
qPhcR-F	TGCTGTCGACCTTCCTGAAT		This study
qPhcR-R	ATCCGGTAGGACACCCAATC	154 bp	This study
qPhcS-F	CCAGCTGAAAGAGGAACTGG		This study

qPhcS-R	AGAAGTTGACGGGGTTGTTG	161 bp	This study
qXpsR-F	AGATCGACATAGCGCTGCTT		This study
qXpsR-R	TTACTTTGCGGACCTGCTCT	150 bp	This study
qEpsE-F	CTGGATAAAGCCACGCAAAG		This study
qEpsE-R	CAGTGGTACATCGCCATCAC	202 bp	This study
