# Supplementary figures and images for: Oleanolic Acid Induces the Type III Secretion System of Ralstonia solanacearum
Source: Front Microbiol. 2015 Dec 22;6:1466. doi: 10.3389/fmicb.2015.01466 (PMC4686671; doi:10.3389/fmicb.2015.01466)

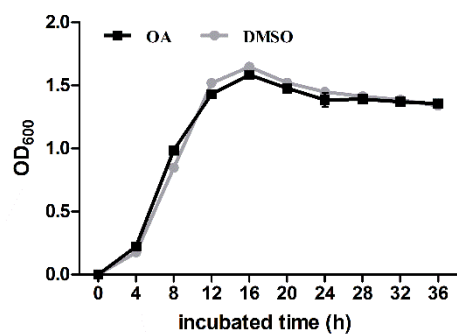

Figure S1. The effect of OA on the growth of *Ralstonia solanacearum*.

Supplement: Supplementary file 2 [file Image_1.PDF]

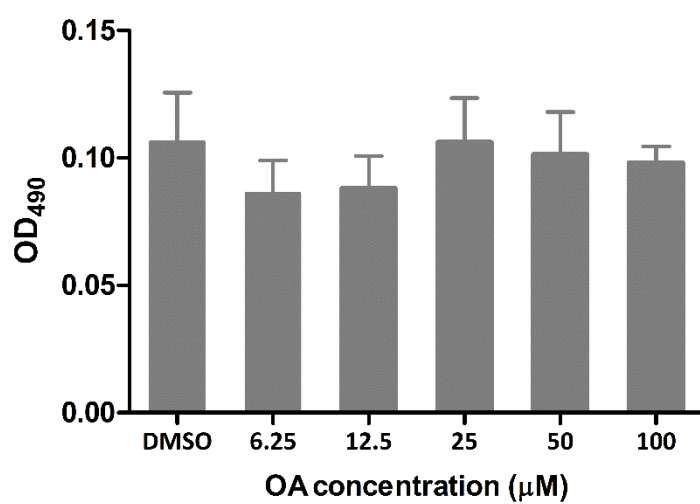

Figure S2. The effect of OA on *R. solanacearum* biofilm formation.

Supplement: Supplementary file 3 [file Image_2.PDF]

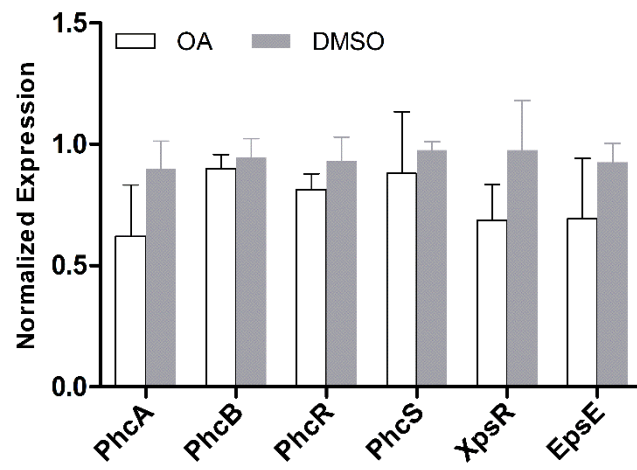

Figure S3. The effect of OA on the expression of some virulence genes in *R. solanacearum*.

Supplement: Supplementary file 4 [file Image_3.PDF]
